# Bilateral Gonadoblastoma With Dysgerminoma in a Phenotypically Normal Female With 46XX Karyotype: Report of a Rare Case and Literature Review

**DOI:** 10.7759/cureus.8990

**Published:** 2020-07-03

**Authors:** Muhammad Abdur Raafey, Muhammad Abdulwaasey, Syeda Samia Fatima, Zeeshan Uddin, Muhammad Usman Tariq

**Affiliations:** 1 Cell and Molecular Medicine, Pathology, Rush University Hospital, Chicago, USA; 2 Histopathology, Pathology and Laboratory Medicine, Aga Khan University Hospital, Karachi, PAK; 3 Pathology and Laboratory Medicine, Aga Khan University Hospital, Karachi, PAK

**Keywords:** gonadoblastoma, dysgerminoma, gonadal dysgenesis, 46xx, karyotype

## Abstract

Gonadoblastoma is a rare ovarian neoplasm which belongs to “germ cell-sex cord-stromal tumor” category. This tumor is frequently associated with invasive germ cell malignancy. It commonly arises in dysgenetic gonads of young individuals who are phenotypically females but possess 46XY karyotype. It has been rarely reported in females with normal phenotype and genotype.

We herein describe a case of 10-year-old female who presented with abdominal pain, abdominal distention and fever. CT scan of the abdomen and pelvis revealed bilateral ovarian masses, ascites and pelvic and para-aortic lymphadenopathy. Serum lactate dehydrogenase levels were also elevated. She underwent left salpingo-oophorectomy, right ovarian biopsy, omentectomy and para-aortic lymphadenopathy.

Microscopically, tumor showed in situ and invasive components. In situ component was arranged in nests and lobules formed by immature sertoli cells forming acini and encircling large polygonal primitive germ cells. Immature sertoli cells were positive for immunohitochemical (IHC) stains cytokeratin AE1/AE3, inhibin and calretinin, while germ cells were positive for SALL4, Oct 3/4, placental alkaline phosphatase (PLAP) and CD117. Invasive component was arranged in sheets of large-sized, polygonal-shaped primitive germ cells which were also positive for SALL4, Oct 3/4, PLAP and CD117 IHC stains. Hence, the diagnosis of “gonadoblastoma with dysgerminoma” was made. The tumor was limited to both ovaries. Cytogenetic analysis of peripheral blood revealed normal female 46XX karyotype. The patient received two cycles of adjuvant chemotherapy and was then lost to follow-up.

We conclude that gonadoblastoma, although rare, should be considered as a differential diagnosis in ovarian tumors of young females. Invasive germ cell component should always be carefully searched for as it guides about treatment and predicts prognosis.

## Introduction

Gonadoblastoma is a rare ovarian neoplasm that comprises a mixture of primitive germ cells and immature sex cord cells [1,2]. It is considered an “in situ” germ cell malignancy that can be associated with “invasive” germ cell neoplasms [1,3]. It is mostly detected in young females during evaluation for amenorrhea and malformed female genital tract [1]. Virilization is a common symptom that occurs due to production of testosterone and estrogen by these tumors [1,2,4]. Radiologically, these tumors present with adnexal calcifications which results in gritty cut surface of the tumor on gross examination [1]. Microscopically, tumor is composed of acini formed by sex cord-type (immature granulosa and sertoli) cells arranged around primitive germ cells [1,5]. In majority of cases, gonadoblastomas are associated with malignant germ cell tumors, typically dysgerminoma or occasionally immature teratoma, yolk sac tumor, embryonal carcinoma or choriocarcinoma [1,6,7]. The presence of elevated serum markers, such as lactate dehydrogenase (LDH), β-human chorionic gonadotropin (β-HCG) and alpha fetoprotein (AFP), may complicate the clinical diagnosis in these tumors [2,7].

Most of the patients are phenotypic females with gonadal dysgenesis and 46XY karyotype. However, abnormal gonads may not the rule and rare cases of phenotypically and genotypically normal females, i.e. with 46XX karyotype and normal gonads, have also been reported [1,6].

Management of gonadoblastoma essentially consists of surgery that may be followed by chemotherapy in cases with malignant germ cell component. Prognosis of gonadoblastoma depends on the characteristics of malignant germ cell component. However, cases with dysgerminoma have also reported excellent outcome [6,8].

We herein report clinicopathological features of an extremely rare case of bilateral gonadoblastoma with dysgerminoma in a young female with phenotypically normal female genital tract and normal 46XX karyotype.

## Case presentation

A 10-year-old female presented to the gynecology clinic with complaints of abdominal pain, abdominal distention and fever for 15 days. On physical examination, generalized abdominal tenderness, distention and ascites were noted. Rest of the physical examination was unremarkable, and normal female external genitalia were noted. CT scan of the abdomen and pelvis revealed a large solid left adnexal mass with specks of calcifications measuring 16 x 10 x 9 cm. Right ovary was also mildly enlarged with calcifications and measured 3 x 1 x 0.5 cm. Both fallopian tubes, uterus and vagina were well formed and appeared unremarkable except for hydrometrocolpos. Ascites and multiple enlarged para-aortic and retroperitoneal lymph nodes were also noted.

Rest of abdominal and pelvic viscera were unremarkable. No omental or peritoneal deposits were identified. Evaluation of serum tumor markers revealed raised levels of LDH and normal levels of AFP and β-HCG. Based on these findings, exploratory laparotomy, left salpingo-oophorectomy, right ovarian biopsy, para-aortic lymph node sampling and partial omentectomy were performed.

**Figure 1:**
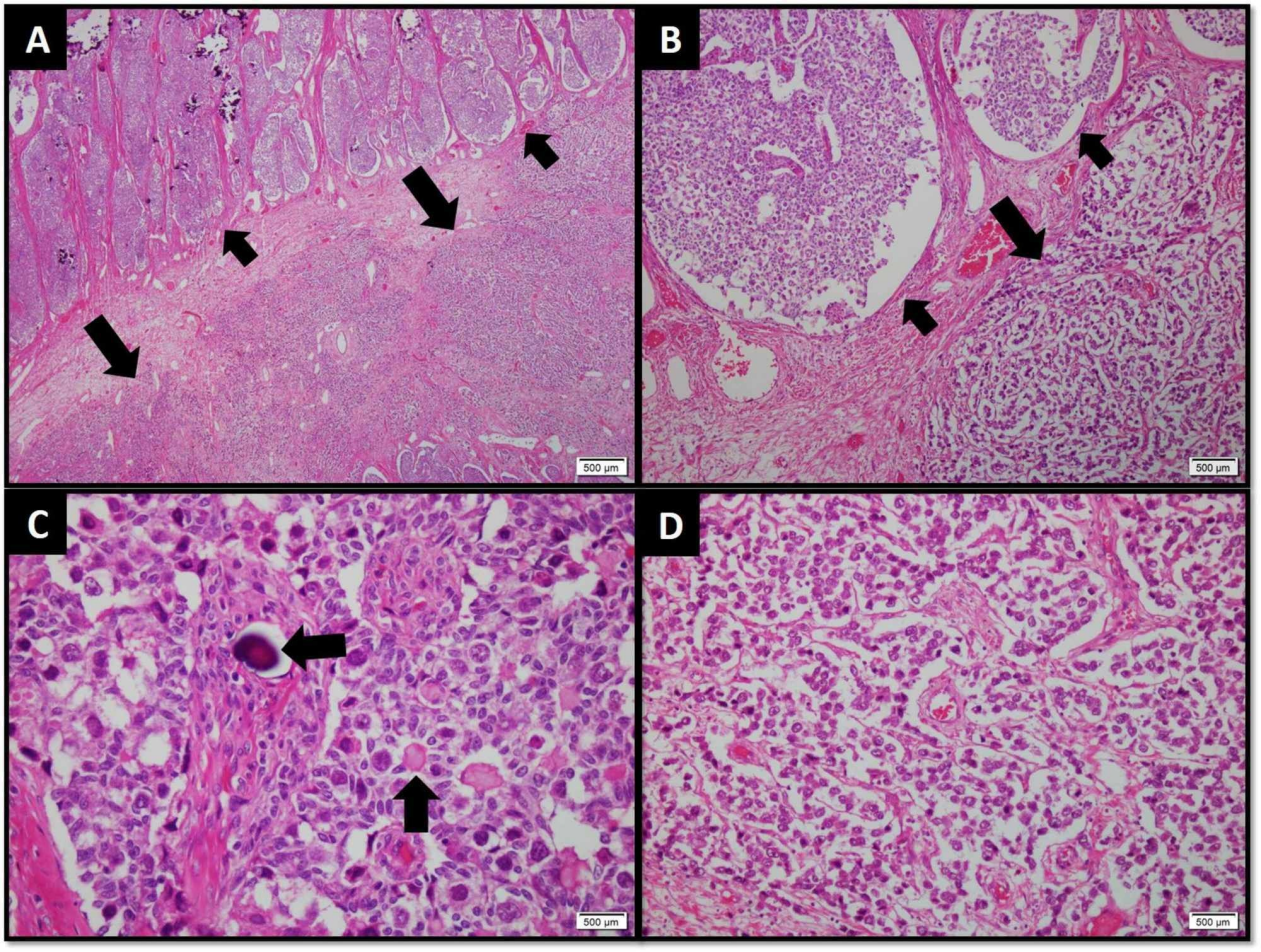
(A) Low-power and (B) medium-power view of tumor exhibiting gonadoblastoma arranged in large nests and lobules (smaller arrows) and dysgerminoma arranged in sheets (larger arrows). (C) High-power view of gonadoblastoma showing cuboidal sex cord-type cells forming acini around larger polygonal primitive germ cells. Calcification (horizontal arrow) and Call-Exner bodies (vertical arrow) are also evident. (D) High-power view of dysgerminoma arranged in sheets with thin fibrovascular septae

Macroscopically, cut surface of the left ovarian mass was lobulated, firm, pale white, solid with few spongy and myxoid areas. Focal calcification was seen. Necrosis was absent. Cut surface of the smaller ovary was firm, pale white, solid and gritty. Microscopic examination of both ovarian masses revealed a neoplastic lesion comprising of germ cell and sex cord-stromal components arranged in sheets along with nests and lobules separated by fibrous septae (Figures 1A, 1B). Calcifications were frequently appreciated. The sex cord component comprised of acini of cuboidal cells containing moderate amount of cytoplasm and oval nuclei with fine chromatin and inconspicuous nucleoli. Foci of cells arranged around acellular hyaline material were seen (Figure 1C). The germ cell component comprised of round to polygonal primitive germ cells with pleomorphic, vesicular nuclei with prominent nucleoli and frequent mitotic figures. These cells were present within the lumen of acini as well as in diffuse sheets (Figures 1C, 1D).

**Figure 2:**
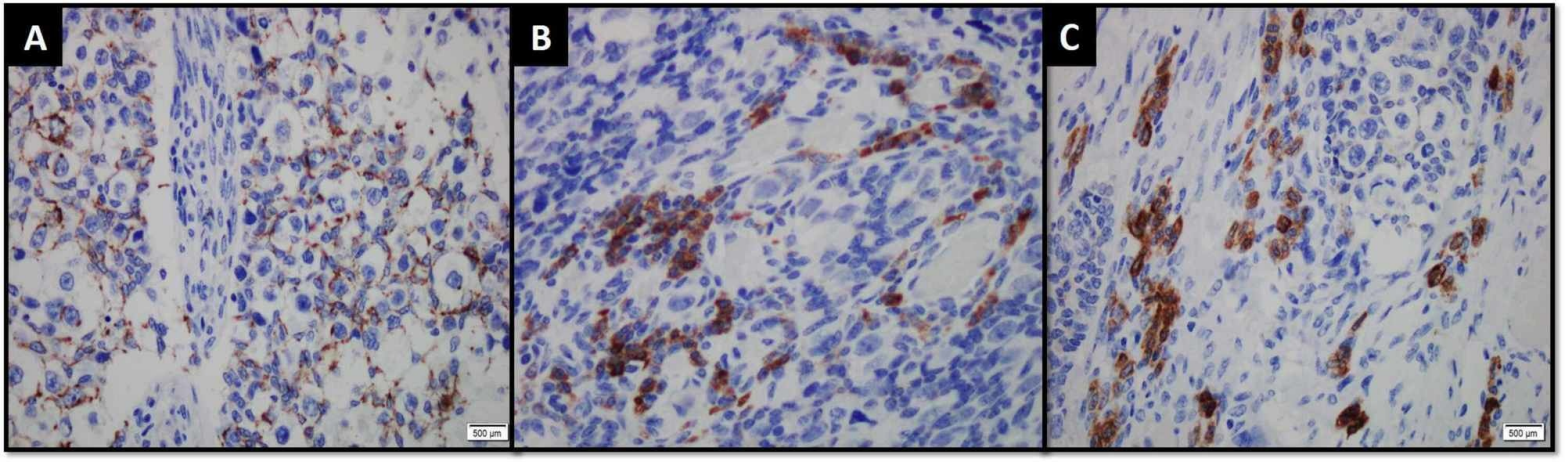
Sex cord component demonstrating positive expression for (A) cytokeratin AE1/AE3, (B) inhbin and (C) calretinin immunohistochemical stains.

The sex cord component demonstrated positive expression for cytokeratin AE1/AE3, inhibin and calretinin immunohistochemical (IHC) stains (Figure 2).

**Figure 3:**
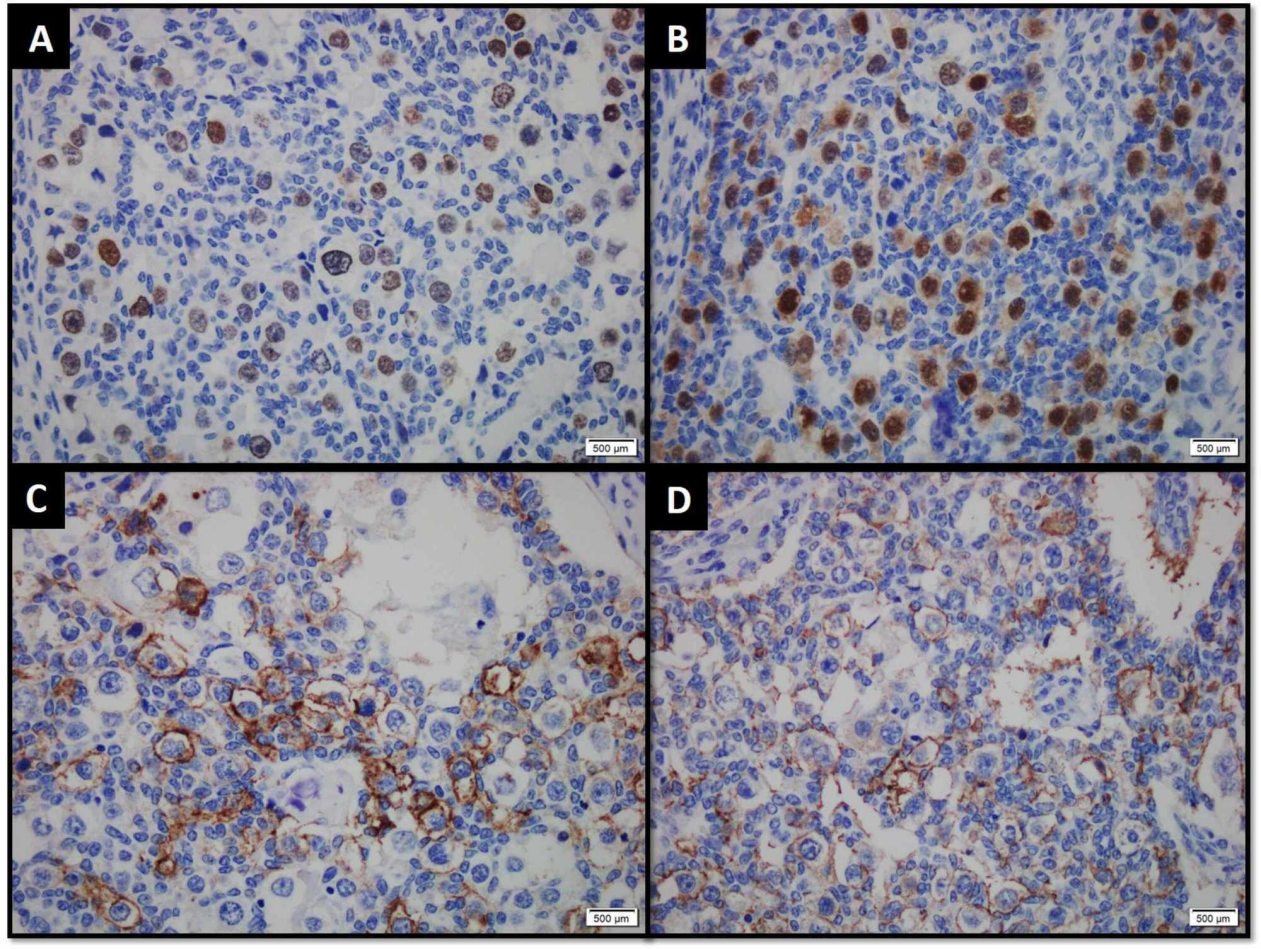
Germ cell component demonstrating positive expression for (A) SALL4, (B) Oct 3/4, (C) placental alkaline phosphatase (PLAP) and (D) CD117 immunohistochemical stains.

SALL4, Oct 3/4, placental alkaline phosphatase (PLAP) and CD117 IHC stains were positive in germ cell component (Figure 3).

Capsule of left ovarian mass was microscopically breached by tumor. Based on these findings, the diagnosis of “gonadoblastoma with dysgerminoma” was made. Left fallopian tube, omentum and para-aortic lymph nodes were tumor free.

Cytogenetic analysis of the peripheral blood revealed normal 46XX karyotype. No Y chromosome was identified. Cytogenetic analysis was not performed on tumor tissue.

Patient received two cycles of chemotherapy (bleomycin, etoposide and cisplatin), and then unfortunately she was lost to follow-up.

## Discussion

Gonadoblastoma commonly occurs in the second and third decades of life with a mean age of 18 years [1]. Pure gonadoblastoma is usually small in size but it may acquire large size due to overgrowth of associated invasive component [2]. Bilateral tumors are observed in more than 40% cases [1,9,10]. Serum tumor markers can be variably elevated in these tumors depending upon the constituent germ cells [1,2,6]. The common symptoms of these tumors include virilization, hirsutism, amenorrhea, abdominal pain, abdominal distention and vaginal bleeding [1,2,4,6]. Histologically, these tumors comprise a combination of sex cord component and primitive germ cell component. The sex cord component morphologically and immunohistochemically resembles immature sertoli and granulosa cells. These cells encircle morphologically and immunohistochemically distinct primitive germ cell component. These cells also surround acellular hyaline material imparting a macrofollicular pattern resembling Call-Exner bodies more commonly associated with adult granulosa cell tumor. Calcifications are frequently observed and provide a useful diagnostic clue [1-3,9]. Careful examination for identification of invasive malignancy is necessary because approximately 50%-60% gonadoblastomas are associated with malignant germ cells tumors which are pure dysgerminoma in most of the cases. Other germ cells tumors including immature teratoma, embryonal carcinomas, yolk sac tumor and choriocarcinomas are rarely observed [1,5,6,11]

Majority of the gonadoblastoma arise in patients genotypically having Y chromosome and phenotypically having dysgenetic gonads [12]. The genotype in most of the cases is 46XY. However, a subset of these patients also exhibits mosaicism and 45X/46XY karyotype [13]. Normal 46XX karyotype is observed in rare cases [6,7,11,14]. To the best of our knowledge, so far only 12 cases of gonadoblastoma with normal female 46XX karyotype have been reported in English literature [2,3,6,7,9,10,11,14].

Cytogenetic analysis/karyotyping of peripheral blood determines the germline status of Y chromosome. In cases of mosaicism, tumor tissue shows the presence of Y chromosome while Y chromosome is absent on karyotyping of peripheral blood. McCuaig et al. performed cytogenetic analysis on peripheral blood, tumor tissue and adjacent ovarian tissue. This suggested that the Y chromosome status should be assessed in tumor tissue and peripheral blood using different techniques such as fluorescence in situ hybridization and quantitative fluorescence-polymerase chain reaction [14]. 

One-third of these cases were bilateral [3,6,9,10]. Around seven cases have been reported in fertile females who have been pregnant [11]. Esin et al. summarized the findings of seven gonadoblastoma cases having normal 46 XX karyotype, reported between 1990 and 2011. The age of these patients ranged from 10 to 27 years. In six out of these seven cases, tumor size ranged from 8 to 22 cm. All of these cases had co-existent invasive germ cell malignancy which was pure dysgerminoma in three cases, dysgerminoma and yolk sac tumor in one case and pure yolk sac tumor and mixed germ cell tumor in one case each. The case described by Esin et al. was incidentally found 0.5 cm gonadoblastoma with dysgerminoma. The patient had concomitant ectopic tubal pregnancy [6]. Kim et al. revealed the findings of two cases of gonadoblastoma with dysgerminoma in 30-year-old and 33-year-old females. The tumor size was 8.5 and 1.8 cm, respectively [3]. Roth et al. shared the experience of a case of nine-year-old female having gonadoblastoma with mixed germ cell tumor comprising of immature teratoma, yolk sac tumor and embryonal carcinoma [7]. Kanagal et al. reported the largest gonadoblastoma with dysgerminoma measuring 30 cm in a 14-year-old female [2]. McCuaig et al. also identified a gonadoblastoma with dysgerminoma measuring 2.5 cm in a 20-year-old female [14]. The findings of our case are in concordance with the findings of other gonadoblastoma cases with normal female phenotype and genotype. Our patient was a 10-year-old female who had bilateral gonadoblastoma with dysgerminoma. Left-sided tumor measured 16 cm and right-sided tumor measured 3 cm in maximum dimension.

Pure gonadoblastomas behave in a benign fashion. Cases with dysgerminoma have excellent prognosis. However, prognosis is unfavorable for other tumor types such as yolk sac tumor [6,8].

Surgery is the mainstay of treatment, and most of the patients undergo oophorectomy which can be accompanied by salpingectomy, hysterectomy, omentectomy and lymph node dissection [2,6,10]. Germline and tumoral Y chromosomal analysis can help in avoiding contralateral oophorectomy especially in young patients [14]. Cases with co-existent invasive malignancy require adjuvant chemotherapy [3,6,10]. The patient in our study underwent left salpingo-oophorectomy, right ovarian biopsy, omentectomy and para-aortic lymph node sampling. The disease was limited to the ovaries. The patient received two cycles of chemotherapy and then she was lost to follow-up.

The lack of follow-up information about our patient is the main limitation of this case report. However, the clinical and histopathological features of our patient are quite characteristic and further endorse the features reported in the literature.

## Conclusions

Gonadoblastoma can occur in young females who are phenotypically and genotypically normal and therefore should be considered as a differential diagnosis in these patients. It is frequently associated with invasive malignancy which is dysgerminoma in most cases. The prognosis is generally excellent. However, careful search for invasive germ cell tumor is necessary as it determines the prognosis and subsequent treatment plan.
